# Supplementary material for: Mapping the evolution of fertility support policies in China: A content and instrumental analysis
Source: PLoS One. 2025 Oct 9;20(10):e0332137. doi: 10.1371/journal.pone.0332137 (PMC12510515; doi:10.1371/journal.pone.0332137)
Supplement: S1 Appendix — (ZIP) [file pone.0332137.s001.zip › S1 Appendix. 226 original policy documents/220-财政部、卫生计生委关于进一步完善计划生育投入机制的意见(FBM-CLI-4-278725).doc]

财政部、卫生计生委关于进一步完善计划生育投入机制的意见

**发布部门：** [财政部](https://www.pkulaw.com/law/chl?Aggs.IssueDepartment=60210) [国家卫生和计划生育委员会(已撤销)](https://www.pkulaw.com/law/chl?Aggs.IssueDepartment=60319) [机构沿革](javascript:void(0))

**发文字号：**财社〔2016〕16号

**发布日期：**2016.04.15

**实施日期：**2016.04.15

**时效性：** [现行有效](https://www.pkulaw.com/law/chl?Aggs.TimelinessDic=01)

**效力级别：** [部门规范性文件](https://www.pkulaw.com/law/chl?Aggs.EffectivenessDic=XE0302)

**法规类别：** [计划生育管理](https://www.pkulaw.com/law/chl?Aggs.Category=09705)

财政部、卫生计生委关于进一步完善计划生育投入机制的意见
（2016年4月15日　财社[2016]16号）

各省、自治区、直辖市、计划单列市财政厅(局)、卫生计生委，新疆生产建设兵团财务局、人口计生委：
　　为贯彻落实党中央、国务院关于实施全面两孩政策、改革完善计划生育服务管理的决策部署，支持统筹推进生育政策、服务管理制度、家庭发展支持体系和治理机制综合改革，促进人口长期均衡发展，现就调整完善计划生育投入机制提出以下意见：
　　**一、**充分认识调整完善计划生育投入机制的重要意义
　　党中央、国务院历来高度重视计划生育工作，《中共中央国务院关于实施全面两孩政策　改革完善计划生育服务管理的决定》明确了稳妥扎实有序实施全面两孩政策、大力提升计划生育服务管理水平、构建有利于计划生育的家庭发展支持体系的目标和任务。
　　当前和今后一段时期，我国人口发展面临新的形势。实行计划生育40多年来，大量独生子女父母逐步进入老龄人口行列，调整计划生育服务管理制度，健全家庭发展体系，意义十分重大，任务十分艰巨。按照党的十八届五中全会关于促进人口均衡发展，坚持计划生育的基本国策，完善人口发展战略，全面实施一对夫妇可生育两个孩子政策，积极开展应对人口老龄化行动等要求，必须进一步完善相关政策措施，建立健全促进人口长期均衡发展的投入机制。

　　**二、**调整完善计划生育投入机制的主要内容
　　按照事权与支出责任相适应的财政体制改革方向，调整完善计划生育投入机制，推动计划生育工作由控制人口数量为主向调控总量、提升素质和优化结构并举转变，由管理为主向更加注重服务家庭转变，由主要依靠政府力量向政府、社会和公民多元共治转变。
　　(一)加大扶助保障力度。国家规定的计划生育扶助保障项目所需经费，由中央和地方按比例安排，补助资金按照目标人群数量和人(户)均标准、绩效评价结果等因素分配。中央财政以2015年补助数为基数，增支部分(包括自然增支和提标增支)对西部地区、中部地区分别按照80％、60％的比例，对东部地区按照50％-10％不同比例予以补助。继续实施农村部分计划生育家庭奖励扶助制度、计划生育家庭特别扶助制度及“少生快富”工程。
　　(二)统一城乡独生子女伤残、死亡家庭扶助标准。完善计划生育家庭特别扶助制度，统一城乡独生子女伤残、死亡家庭扶助标准，将农村独生子女伤残、死亡家庭扶助标准提高到与城镇水平一致。根据经济社会发展水平等因素，实行特别扶助制度扶助标准动态调整。
　　(三)加快独生子女父母奖励费制度改革。按照国家和各地区有关规定落实独生子女父母奖励经费。对全面两孩政策实施后自愿只生育一个子女的夫妻，不再实行独生子女父母奖励优惠等政策。
　　(四)调整经费使用方向，支持改革完善生育服务管理。按照法律、法规和政策规定，调整支出方向，重点支持实行生育登记服务制度、加强出生人口监测预测、综合治理出生人口性别比偏高、加强基层计划生育人员培训、落实计划生育目标管理责任制考核等工作。同时，支持做好常住人口(含流动人口)计划生育、优生优育、技术服务、宣传教育、信息统计等工作。
　　(五)鼓励、引导社会资金投入，建立多渠道筹资机制。鼓励企业、社会团体和个人投入计划生育事业，鼓励按照国家有关规定设立人口和计划生育公益基金。积极运用项目合作机制，吸引国内外资金。

　　**三、**工作要求
　　调整完善计划生育投入机制工作，涉及面广，政策性强。各级财政和卫生计生部门要从全局出发，周密部署，统筹安排，扎扎实实把各项政策贯彻落实到位。
　　(一)加强组织领导，搞好协调配合。各级财政和卫生计生部门要切实加强对调整完善计划生育投入机制工作的组织领导，加强协调，密切配合。省级财政部门要会同卫生计生部门按照本意见的要求，抓紧制定切实可行的实施方案。财政部、卫生计生委将加强对调整完善计划生育投入机制工作的指导和协调。
　　(二)落实筹资责任，确保投入到位。省级财政部门要会同卫生计生部门统筹考虑与相关政策和制度的有效衔接，有效利用医疗卫生资源，增强基层计划生育部门提供公共服务的能力。保障县级卫生计生部门履行管理和服务职能所必须的经费以及各项扶助保障政策、计划生育技术服务等支出。
　　(三)完善考核办法，强化资金管理。各地区要将财政投入纳入计划生育工作目标管理责任制考核。财政部门和卫生计生部门要加强预算编制和预算执行管理，确保资金分配及时，资金使用规范、安全和有效。卫生计生部门要按照财政财务管理有关制度规定，努力提高资金使用效益。财政部和卫生计生委将加强监督检查，实施绩效评价，根据工作开展情况，在安排中央转移支付资金时给予奖惩。

© 北大法宝：（[www.pkulaw.com](https://www.pkulaw.com/)) 专业提供法律信息、法学知识和法律软件领域各类解决方案。北大法宝为您提供丰富的参考资料，正式引用法规条文时请与标准文本核对。
欢迎查看所有[产品和服务](https://www.pkulaw.com/helps/2.html)。法宝快讯：[如何快速找到您需要的检索结果？](https://www.pkulaw.com/helps/70.html)[法宝](https://www.pkulaw.com/helps/69.html)V6有何新特色？

扫描二维码阅读原文

**原文链接：**<https://www.pkulaw.com/chl/a227a1649665857dbdfb.html>
